# Developing relevant community mental health programmes in North India: five questions we ask when co-producing knowledge with experts by experience

**DOI:** 10.1136/bmjgh-2022-011671

**Published:** 2023-08-31

**Authors:** Pooja Pillai, Meenal Rawat, Sumeet Jain, Rachelle Anne Martin, Kakul Shelly, Kaaren Mathias

**Affiliations:** 1 Community Health and Development Program, Herbertpur Christian Hospital, Herbertpur, India; 2 School of Social and Political Science, The University of Edinburgh, Edinburgh, UK; 3 Department of Medicine, University of Otago, Wellington, New Zealand; 4 Faculty of Health, University of Canterbury, Christchurch, New Zealand

**Keywords:** mental health & psychiatry, other study design

## Abstract

Knowledge co-production can improve the quality and accessibility of health, and also benefit service users, allowing them to be recognised as skilled and capable. Yet despite these clear benefits, there are inherent challenges in the power relations of co-production, particularly when experts by experience (EBE) are structurally disadvantaged in communication skills or literacy. The processes of how knowledge is co-produced and negotiated are seldom described. This paper aims to describe processes of co-production building on the experiences of EBE (people with lived experience of psychosocial or physical disability), practitioners and researchers working together with a non-profit community mental health programme in North India. We describe processes of group formation, relationship building, reflexive discussion and negotiation over a 7-year period with six diverse EBE groups. Through a process of discussion and review, we propose these five questions which may optimise co-production processes in communities: (1) Who is included in co-production? (2) How can we optimise participation by people with diverse sociodemographic identities? (3) How do we build relationships of trust within EBE groups? (4) How can we combine psychosocial support and knowledge co-production agendas in groups? and (5) How is the expertise of experts by experience acknowledged?

**Summary Box**

We propose five questions to consider in developing a transparent and collaborative partnership between researchers and people with lived experience:

1. Who is included in co-production?

2. How can we optimise participation by people with diverse socio-demographic identities?

3. How do we build relationships of trust within EBE groups?

4. How can we combine psychosocial support and knowledge co-production agendas in groups?

5. How is the expertise of Experts by Experience acknowledged?

- Co-production can increase ownership, self-determination, and choice for people typically on the receiving end of healthcare.
- Co-production can improve the quality of services and programmes, so that they are more contextually relevant and acceptable.
- Acknowledging the expertise of EBE requires both social and material resources.
- The processes of co-production are as important as the products, and require active, attentive, and reflexive participation by all.

## Introduction

Co-production aims to actively involve those traditionally on the receiving end of health services and research, to partner in designing, implementing, delivering and evaluating relevant care, resources and services.^1^ It offers the potential to open up an opportunity for a ‘third space’ where the expert knowledge of the professional and the expert experience of the service user and carer can enable negotiation of meaning and representation.^2^ Co-productive methods are appreciated for their potential to ensure that services are person-centred, cost-efficient, innovative and equitable.^3–5^ This paper was developed by practitioners, academics and people with lived experience of mental distress, critically reflecting on their experiences and the journey of co-production of resources and services within a North Indian community mental health initiative, Burans, which was started in 2014 and is administered by Herbertpur Christian Hospital. This initiative has sought to co-produce programmes and interventions with people with lived experience of mental distress (experts by experience—EBE) in urban and rural communities and has critically reflected on these processes and how they, both achieved and failed to achieve genuine equal sharing of power.^6–8^ Recognising that power dynamics and privileged sites of knowledge production can limit participation of the most disadvantaged,^9 10^ we sought to consider the practical approaches we used to ensure that appropriate epistemological and power concerns were addressed when engaging in co-production practices. Co-production groups included representation and involvement of people experiencing multiple axes of poverty, social exclusion, and disadvantage.

Co-production reduces the ‘relevance gap’ often encountered when academics from a different context develop interventions and lack lived experience.^11 12^ Furthermore, co-production in disadvantaged communities also benefits the users, allowing them to be seen as skilled, capable and experienced.^13^ This shift in positioning can democratise the process and shift the long-term power imbalance in favour of people with lived experience, rather than in favour of professionals and people with formal tertiary education.^14^

Yet while there is compelling evidence for co-production, the ways that it happens (processes) are also important to examine. The structures established by biomedicine and colonisation mean that ‘the question of what counts as knowledge and whose knowledge counts are fundamentally crossed by questions of power and privilege’.^15^ People with psychosocial disabilities are typically approached as lacking reason, and their knowledge is inferiorised to suggest they are not fit to address their own health needs. Thus the ‘expertise’ of external service providers is considered requisite to develop community health programmes which can add to a sense of inadequacy among community members. Participation in mental health knowledge production for programmes or policies is extremely limited in South Asia, and most markedly for people living in disadvantaged settings.^3 16 17^

This marginalisation from knowledge production is even more evident for those living in low-income settings, for example, where literacy and education have been less accessible.^18^ The under-representation of people experiencing psychosocial disability and who live in poverty limits the relevance and availability of services and resources to support their socio-economic development.^9^

There is a spectrum in the types of participation,^19^ meaning co-production can also veer toward tokenism. For example, while a health service provider might ask ‘What matters to you?’ they may exclusively seek perspectives on a specified service, without being willing to address concerns about wider health and social systems that discriminate and exclude people with psychosocial disability.^20^ People with psychosocial disabilities have challenged this framing and argued that co-production must engage with privilege and the established history of ideas perpetuated by that privilege.^20^

And beyond the contestation about who participates in knowledge production, there is also contestation of the philosophical practices and pragmatic processes used in co-production. For instance, it has been argued that co-production uses methods favouring those with greater social advantage, such as white people and men.^10 12 13^ Privileging some perspectives can lead to biased outcomes not reflective of every participating member.^21^ Co-production has also been criticised for being time-consuming and expensive compared with other alternatives.^21^

Processes used to form partnerships with community members with psychosocial disability, including how knowledge is co-produced and negotiated, are seldom described,^22^ and can point others to processes that can allow them to pre-emptively avoid the pitfalls and dead-ends of co-production. This can strengthen the practice of co-production by others and increase the possibility that it is reflexive, creative and humble.^9^

This paper builds on the framework of participatory action research which uses a cyclical and spiral process of systematising experience, collectively analysing and problematising, reflecting on and choosing action, taking and evaluating action and systematising learnings.^14^ Building on this process, we developed five questions to consider in developing a transparent and collaborative partnership between researchers and people with lived experience, by reflecting on our co-production experiences as community mental health practitioners working in a disadvantaged low/middle-income country setting (MR, PP, KM), with reflexive discussion with an expert by experience group member (KS) and researchers working in the area of co-production (RAM, SJ).

## Context

Co-authors had identified a ‘data gap’ of how co-production is done in practice, especially when including people who are intersectionally disadvantaged and resolved to meet together to critically reflect on the co-production processes we had used, discuss shortcomings and mistakes, synergies, learnings and challenges to in some measure address this gap. The practitioner team met monthly over 9 months, and we held four meetings with the EBE group member and researchers during this time. Initial discussions were free-flowing, using a reflexive process when sharing examples and discussing what worked well and what did not (systematising learnings and collectively analysing and problematising in the Participatory Action Research (PAR) framework).^14^ While in an earlier draft, we had followed Tuckman’s team development framework (ie, norming, forming, storming, performing),^23^ following peer reviewer feedback we recognised that the Tuckman’s framework origins in Euro-American organisational development did not fully fit the context and process. Instead, we returned to the PAR framework we had used in our first account of co-production. The questions we identified paid attention to power relations and the PAR framework we had used for that first description of co-production process.^6^

Data used in this paper included notes and transcripts from co-production group meetings, and our own experiences participating in co-production. We analysed the process of co-production chronologically to form the sequence of ‘questions’ outlined in the Findings and Discussions section. Verbatim quotes presented are taken from recordings and notes taken during meetings exclusively from Groups A and B. Ethics permission for Group A in 2017, with protocol number 143, and for Group B in 2018, with Protocol number 180, were granted by the Institutional Ethics Committee of the Emmanuel Hospital Association.

### Expert by experience group details

Table 1 outlines the six co-production groups the co-authors worked with, linked to Burans, each formed for different purposes. Most group members were women, reflecting gender norms of availability during working hours in northern India. A typical meeting involved group members checking in on each other, a team building or ice breaker activity led by a group facilitator, a recap of the last meeting and then facilitated discussions around the area of knowledge for co-production (such as an upcoming Caregiver intervention project). A team member took hand-written notes and sometimes recorded the meeting for documentation purposes.

**Table 1:** Summary of six EBE groups that have co-produced knowledge and resources with Burans’ teams

	Year group formed	Total members	Participant profile		Duration of group	Group purpose
			Women (n)	Men (n)		
Group A	2017	8	7	1	6 months	Co-developing Swasthya Labh Saadan (SLS) pictorial recovery tool^7^
Group B	2018	7	6	1	6 months	Advisory body to understand the role of EBE groups
Group C	2019	8	8	0	2 years	Advisors to Burans programmes implemented in communities, to understand local needs and assets in new field location
Group D	2018	4	0	4	8 weeks	Youth advisory body for Nae Disha (a youth resilience programme) adaptation
Group E	2020	12	12	0	10 weeks	Youth advisory body for funding proposal development
Group F	2019	11	5	6	Currently active	Advisory body to understand local needs and resources in Buran’s new target location

EBE, experts by experience.

## Five questions for co-production

**Figure 1:**
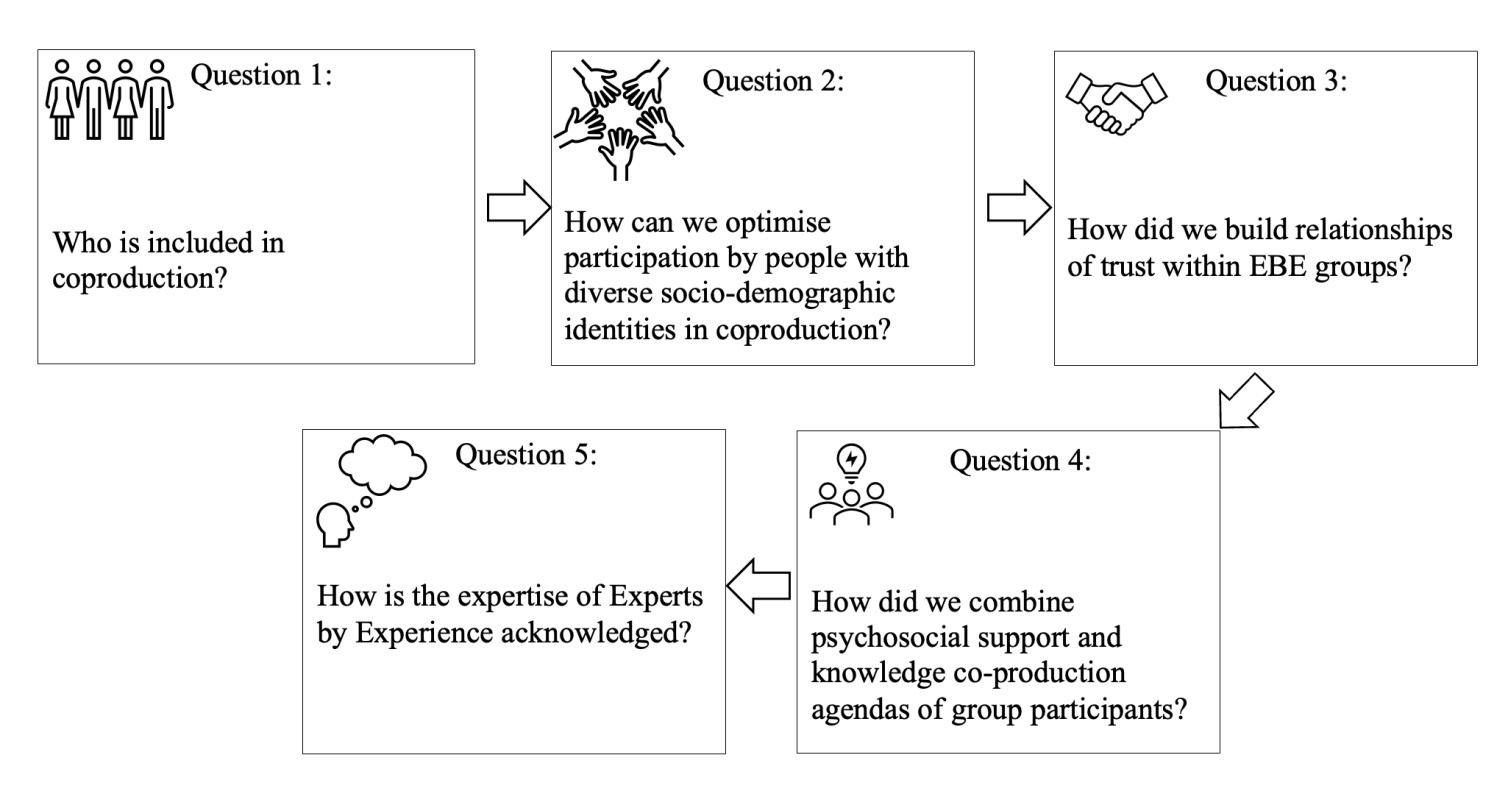
An overview of key learnings from different stages involved in developing an active EBE group. EBE, experts by experience.

We identified five questions that might strengthen the practices of co-production which are outlined in figure 1.

### Question 1: Who is included in co-production?

We sought to ensure representation of diverse identities in EBE groups which in particular included people with experience of structural disadvantage, to ensure outputs that promoted equitable processes and outcomes. To do this, Burans community health workers invited participation by community members with at least two of the following intersectional identities: (1) location of their residence (either informal urban communities or remote rural villages), (2) lived experience of psychosocial disability, (3) limited education, (4) female gender, (5) living in poverty and (6) female head of household. Participation for individuals was often contingent on pragmatic considerations, such as their availability to meet during the working day and feeling able to express an opinion in a group setting. A regular meeting time and location were mutually agreed by EBE and practitioners in the first meeting, along with remuneration and group expectations and purpose. Some groups remained under-represented, for example, men, who were often engaged in income generation during the daytime meetings.

Group members brought their diverse social, economic and health experiences to the knowledge co-production process. This brought led to a sense of solidarity across different social identities, for example, when caregivers from different castes found that they had shared experiences of social exclusion. Diverse group membership also improved the relevance and generalisability of the outputs, such as Group A members from a Muslim background identified that to use a mental health recovery tool, they would not display pictures of people on the wall but would use them in a folder. Another group member who was not literate suggested using coloured pencils to colour in icons for the same mental health recovery tool, which was also adopted by older people and children to participate in recovery activities. Producing knowledge that is designed for local contexts is practiced best when diverse socioeconomic intersectional identities participate.^9 15 20^

### Question 2: How can we optimise participation by people with diverse sociodemographic identities in co-production?

When groups included a mix of different religions, economic backgrounds and mental health problems, there was possibility to understand each other’s experiences, develop creative solutions that were relevant for co-producing knowledge and to co-produce more generalisable outputs. Initially, we uncritically pursued knowledge co-production tasks, but we soon realised we needed to acknowledge and respond to group members’ needs which led to a greater sense of trust and ultimately increased participation. For example, Group A supported a woman experiencing domestic violence to access legal aid and group members supported her with childcare. Responsiveness to intersectional identities has been identified as central to compassionate and engaged global health initiatives.^24^

While most groups had identified that a ‘good’ group would ensure all members were sharing, in reality, most groups were dominated by a few talkative members and people diagnosed with severe mental health problems and/or low literacy often were very quiet, saying that they were not used to contributing their views at home either. To increase participation facilitators invited group members to speak up in turn, on simple matters, for example, turn-taking to share something they felt thankful for and then moving to take turns expressing opinions on more nuanced aspects of programme design. A further strategy to optimise participation was to seek a group member to contribute in an area that was clearly their expertise. For example, seeking advice from Muslim women about cultural norms such as ‘Which of these pictures is best to show a young woman praying Namaaz (a Muslim ritual prayer)?’. Underlining this expertise increased participation in other areas of co-production.

Yet, while groups could build connections through heterogeneous identities, participants were more likely to engage when they felt they shared a similar socioeconomic background as outlined below:

If we are from similar circumstances, we can understand each other better – if you bring someone from a rich family into this group, they may not understand our problems (Babli, EBE group B member)

In some instances, group spaces provided a safer space for participation when there was greater homogeneity. For example, after several times in Group A when caregivers spoke negatively about family members with mental health problems, people with mental health problems elected to form a separate group from the caregiver group. While Palmer *et al* described that the process of building a safe space occurred while co-producing knowledge together,^25^ in these groups, we found that we first needed a level of mutual relationship and solidarity among all members, which has been identified as key to effective co-production elsewhere.^20^

### Question 3: How did we build relationships of trust within EBE groups?

The development of trust in each group was central to the process of co-production. To build trusting relationships meant the groups met multiple times over 4–8 weeks before starting on the group’s co-production work (phase 0 in co-production as described by Burgess *et al*).^20^ In these first meetings, the group participated in a series of activities including sharing life experiences, games and role plays. Group facilitators shared their own experiences of disadvantage or mental distress and which other group members felt helped them feel able to share their own experiences thereby forming an ‘alliance’.^4^ Groups also showed care and solidarity for each other, for example, a member of Group B shared her fear of losing custody of her child during her divorce. The whole group requested support to learn more about divorce law and women’s rights. Community health workers as co-facilitators also increased a sense of safety in the group in their culturally appropriate understanding of the context which has been described as key to building relationships of trust in diverse settings globally.^26^

Other factors that strengthened trust building included working through differences in opinion and negotiating solutions,^12^ holding meetings held outside the community region, which also provided a more neutral platform.^17^ and the group self-governing where possible, for example, in choosing the time and place of meetings, by setting timelines for co-production outputs and choosing their own name. For example, Group E named themselves ‘Smart Girls’ which increased their sense of connection and belonging.

### Question 4: How did we combine psychosocial support and knowledge co-production agendas of group participants?

In a setting with limited mental healthcare access, mutual psychosocial support emerged as a key benefit of group membership. Others have also identified the value of psychosocial resources to effective co-production^20 25^ and EBE described the group as a ‘safe social space’, a key component of community mental health competency.^27 28^ Group members also described that the psychosocial support aspect of group participation was important to their ongoing participation:

I liked playing with the balloons that day, and just coming out of the house and having a free mind. I enjoyed being a part of this group and meeting everyone (Binita, EBE group A member)

The group was also seen as a key resource for psychosocial support, for example, a group facilitator described how a group member experiencing violence at home was supported by other group members:

Dipika was unusually quiet today.… she started crying and sobbing and talked about how the situation is very bad in her house with her husband drinking and hitting her every day and she is living in absolute fear… she is afraid that she will lose her children…. the entire group was supporting Dipika and two offered to help with childcare…. (Facilitator notes from EBE group B)

After initially making the mistake of trying to focus only on knowledge co-production, group facilitators described how they could play a crucial role in supporting both the advisory and support roles of the group by responding to psychosocial needs as well as receiving co-production advice from the group.^6^ This could raise challenges when the group was trying to co-produce a knowledge output to a research project timeline, but then needing to pause to engage with challenges with home situations of the group. When required, the facilitator made notes of concerns and discussed how they could be addressed at a separate support group meeting.^25^ Setting clear agendas together at the beginning of group sessions helped create boundaries between the group’s psychosocial support and co-productive roles and one group (Group A) agreed to hold alternate meetings with agendas of ‘support’ and ‘coproduction’. The sense of alliance, solidarity and strength of a group that was co-producing together, also strengthened their sense of capability and partnership to address difficult social situations of group members, highlighting some of the beneficial mechanisms of co-production described by Palmer *et al.*^25^

### Question 5: How is the expertise of experts by experience acknowledged?

Group members who had experienced multiple forms of structural disadvantage found it challenging to recognise their own expertise, especially when they had had no previous opportunities to share their opinions publicly.^13^ Group members with limited literacy particularly undervalued their own expertise and ability to contribute:

I was not able to talk in front of everyone as I thought I will say something wrong and embarrass myself. I am illiterate and do not know what to say (Rimjhim, EBE Group B).

Attentive and supportive facilitation was needed to support people with mental distress and limited education as important contributors in the co-production process.^13^ Setting a concrete task that required strong contextual knowledge was one approach that made this expertise more clearly evident. For example, when members of Group A, reviewed the drawings of a middle-class North Indian illustrator, they immediately identified how images could be more locally accurate.^6^

This woman is not wearing a bindi or bangles. How will we know whether she is married or not? Is she a widow? In our culture, these symbols are very important (EBE group A member)

Key to recognising expertise included providing ways of engaging and sharing knowledge that did not use written text. For example, kidney beans were used as ‘votes’ and picture drawings or symbols cut from magazines were used to suggest ideas. Girls in Group E also liked to use voice messages on Whatsapp (a social media app used very widely across India) groups to capture and expand group knowledge production.^13 14 29^ Participating in diverse ways also opened up a safe space for group members to discuss and negotiate which increased their sense of expertise without feeling judged.^30^

Group F took the initiative to implement local World Mental Mental Health Day celebrations across the district, and group members spoke publicly in front of their communities which provided status and acknowledgement of expertise. A further way to recognise expertise was by providing financial recognition for the time of group members. Given the attention to reciprocity and power-sharing within EBE groups, members wanted to discuss the types of benefits they and their represented communities should receive. By discussing what resources that would support group participation, and providing financial remuneration, co-production group members felt that their time and contributions were valued. Remuneration made it easier for women to obtain permission to attend meetings (in the patriarchal setting of North India, this was required for some groups). One group member describes this below:

We are benefitting from this group. We are learning something new. This is why I said from the beginning that you need not give us money. However, I have something to show my husband for the time I spend away from home. It is such a pleasure to be able to buy my child a chocolate from this money (Babli, EBE group C member).

While some EBE members declined payment because they believed they were not experts, that they were benefitting from being part of the group or considered payment a ‘handout’, remuneration helped others feel valued.^31^

Accountability and recognition of co-production was also important in disseminating outputs of co-production. For example, in using a co-produced mental health recovery tool, practitioners described the role of the EBE group and named group members to underline that health advancement in the communities is supported by the co-production of knowledge and that they were also being held accountable for their research praxis.^32 33^

## Implications

In settings with limited mental healthcare resources and diverse populations, co-production can increase ownership, self-determination and choice for people typically on the receiving end of healthcare. In all settings, co-production can also improve the quality of tools and programmes, so that they are more contextually relevant and acceptable for those who use services.

In settings with limited resources and access to formal care, we note there are important beneficial mechanisms triggered for those who are members of a psychosocially supportive group: these include a sense of inclusion, mutual social support, improved skills and knowledge, a sense of belonging and collective strength meaning group members are better able to advocate for their own well-being and address upstream social health determinants. These intersect notably with mechanisms of co-production identified elsewhere, as recognition, dialogue, cooperation, accountability, mobilisation, enactment, creativity and attainment.^25^ When triggered, these mechanisms lead to improved mental health and social participation outcomes.^34^ These mechanisms are recognisable in the processes described in this paper and in a recent study completed by co-authors in this paper, where using a participatory health needs assessment process among communities in a remote rural setting identified multiple assets and resources which could be leveraged to increase social support and mental health even in areas with no formal health service accessible.^35^ Groups that co-produce knowledge can also provide psychosocial support, an approach that can recognise and build on existing assets in communities.

Our experience also clarified that groups operating for more extended periods with a clear agenda and tangible results, were more productive and fruitful, and allowed a greater possibility of transfer of power. The importance of duration of time has been identified in other descriptions of co-production processes.^20^ We note, however, that most of the groups we described here operated for a time period that was too short to realise this benefit, and instead, the socioeconomic realities of group members’ contexts (needing full-time employment, time poverty and wanting to be part of a group with a primary focus on psychosocial support) meant that group members elected not to continue working together in co-production. When co-producing with EBE, setting agendas and timelines are useful, but facilitators also need to consider the unique needs of the context and the specific groups and to consider what conditions a group operates under that will allow a lengthier period of participation.

## Conclusions

There is now widespread recognition that co-production is required to ensure mental health interventions and programmes are high quality, contextually valid and relevant. Co-production is also important for EBE to locate them as experts, provide a platform for creativity, self-determination and participation, and to provide mutual psychosocial resources between group members. Descriptions of the processes of co-production are required to build competencies in co-production for mental health practitioners, to ensure that co-production is authentic, participatory and ultimately transformative.

This paper outlines aspects of the co-production process that have emerged as key in the North Indian setting under the rubric of five questions that we ask ourselves. These include the need for diverse representation from communities to include those who are most structurally disadvantaged, co-production processes that are inclusive of all group members and provide safe social spaces, establishing groups where there are relationships of trust, and providing pragmatic responses to group needs particularly in settings with limited mental healthcare resources. Acknowledging the expertise of EBE is central and requires both social and material resources. The processes of co-production are as important as the products, and require active, attentive and reflexive participation and approaches by all participants.

## Data Availability

Data sharing not applicable as no datasets generated and/or analysed for this study.
